# Association of the Qualitative Clock Drawing Test with Progression to Dementia in Non-Demented Older Adults

**DOI:** 10.3390/jcm9092850

**Published:** 2020-09-03

**Authors:** Hiroyuki Umegaki, Yusuke Suzuki, Yosuke Yamada, Hitoshi Komiya, Kazuhisa Watanabe, Masaaki Nagae, Masafumi Kuzuya

**Affiliations:** 1 Department of Community Healthcare and Geriatrics, Nagoya University Graduate School of Medicine, 65 Tsuruma-cho, Showa-ku, Nagoya, Aichi 466-8550, Japan; yoyamada@med.nagoya-u.ac.jp (Y.Y.); h-komiya@med.nagoya-u.ac.jp (H.K.); n910eb@gmail.com (K.W.); m-nagae@med.nagoya-u.ac.jp (M.N.); kuzuya@med.nagoya-u.ac.jp (M.K.); 2 Centre for Community Liaison and Patient Consultations, Nagoya University Hospital, 65 Tsuruma-cho, Showa-ku, Nagoya, Aichi 466-8560, Japan; yus@med.nagoya-u.ac.jp

**Keywords:** Alzheimer’s disease, clock drawing test, memory clinic, mild cognitive impairment

## Abstract

To evaluate the predictability of progression of cognitive impairment to dementia using qualitative clock drawing test (CDT) scores, we administered both the CDT using Cahn et al.’s qualitative scoring system and the Mini-Mental State Examination (MMSE) to assess cognitive function in non-demented older individuals attending a memory clinic at a university hospital. Patients visiting the clinic for assessment of cognitive function between January 2015 and December 2019 were enrolled, and only those who were diagnosed as not having dementia at the time of initial assessment completed a follow-up assessment at 1 y (*n* = 163). To examine any association of qualitative CDT score with progression to dementia, multiple logistic regression analysis was conducted with the change in diagnosis from non-dementia to dementia at 1 y as the dependent variable. A total of 26 participants (16.0%) were diagnosed as having converted to dementia. Multiple logistic regression analysis revealed that both the qualitative CDT score using Cahn et al.’s scoring system and the existence of conceptual deficits were significantly associated with progression to dementia at 1 y after initial assessment of cognitive function, irrespective of the MMSE score, among non-demented older individuals. The CDT may be a useful predictor of progression to dementia in primary care settings.

## 1. Introduction

As populations age rapidly worldwide, there is considerable clinical interest in accurately diagnosing dementia before it develops, and particularly predicting the subsequent development of dementia among individuals with mild cognitive impairment (MCI). Identifying dementia before or in the early stage has significance not only because this can help to maximize the effects of anti-dementia medications, but also because it could mitigate the impact of actual diagnosis by allowing patients and their families to anticipate and prepare for later difficulties [1]. In addition, alongside early pharmacotherapeutic interventions, many lifestyle-related risk factors such as smoking, depression, social isolation, and physical inactivity have been identified [2], and preventive or interventional approaches have been proposed to help reduce the risk of developing dementia or slow its progression. However, the effect sizes of such approaches are not as large as hoped for, and therefore early identification, preferably in the pre-dementia stage, is desirable.

To help with prediction, several clinical markers for dementia have been established. These include the biomarkers amyloid beta or tau protein in the cerebrospinal fluid (CSF) and amyloid and tau findings on positron emission tomography (PET) neuroimaging [3]. However, these markers are not always available to use in primary care settings because of the high cost involved and the need for specialized equipment or invasive procedures.

The clock drawing test (CDT) is a well-established screening instrument for cognitive impairment. Similar to the Mini-Mental State Examination (MMSE), another commonly used screening test of cognitive function, the CDT is easy to administer, requires no special equipment, and can be administered relatively quickly. The CDT is thus appropriate for cognitive screening in primary care settings. Many studies have reported the utility of the CDT for diagnosing dementia [4,5], and several studies have also reported its usefulness for detecting MCI [6,7]. The CDT is also used to follow the trajectories of cognitive decline in older populations, and some studies have reported the predictability of progression to dementia with its use in non-demented participants [8].

Various methods using the CDT to evaluate cognitive function have been proposed [9]. Among them, the method proposed by Cahn et al., which assesses the drawn clock both qualitatively and quantitatively, has shown good validity, and our previous study showed that it can distinguish MCI from dementia with acceptable certainty [10,11]. Other studies have suggested the usefulness of quantitative CDT scoring for predicting progression to dementia in non-demented populations [12,13], but few have examined the predictability of qualitative scoring for predicting progression to dementia in non-demented individuals [14]. Moreover, none have investigated the usefulness of the Cahn et al. scoring system for predicting such progression.

In this study, to evaluate the predictability of progression from non-dementia status (MCI and cognitively normal) to dementia based on qualitative CDT scores, we administered the CDT using Cahn et al.’s qualitative scoring system and the MMSE to older non-demented individuals attending a memory clinic at a university hospital. We also compared the predictability of the CDT and MMSE, and attempted to combine the administration of these two tests, which was easy to do in the clinical setting.

## 2. Materials and Methods

### 2.1. Participants

Consecutive patients visiting the geriatric department outpatient clinic at Nagoya University Hospital for the assessment of cognitive function between January 2015 and December 2019 were enrolled in the study. Data were collected from medical charts retrospectively. Participants without a diagnosis of dementia or major psychiatric disease, such as major depression and schizophrenia, at the first assessment were included. Participants who did not have a second assessment 1 year later (365 ± 120 days) were excluded from the analysis.

The study protocol was thoroughly reviewed before being approved by the Ethics Committee of Nagoya University Graduate School of Medicine (2020-0071).

### 2.2. Neuropsychological Assessments

A battery of neuropsychological assessments was performed by 3 well-trained clinical neuropsychologists. The assessments were as follows: the MMSE [15] for general screening of cognition; the Logical Memory II subtest of the Wechsler Memory Scale-Revised (WMS-R) [16] and the Alzheimer’s Disease Assessment Scale (ADAS) [17] for evaluation of mnemonic functions; and the Logical Memory I subtests and Visual Reproduction I and II subtests of the WMS-R [18] for evaluation of other cognitive domains. For the assessment of verbal fluency, we used animal naming for categorical fluency as well as the letter fluency test. The Digit Span subtest (forward and backward) and the Visual Memory Span subtest (forward and backward) of the WMS-R were used to assess working memory. The Digit Symbol subtest of the Wechsler Adult Intelligence Scale-III (WAIS-III) [19] was used to evaluate mental processing speed, and the Trail Making Test parts A and B [20] and the Stroop test [21] were used to evaluate executive function. Longitudinal follow-up using the same neuropsychological assessments was performed around 1 year later (mean and standard deviation, 365 ± 120 days) at the discretion of doctors. Only those patients who were diagnosed as not having dementia at the time of the initial assessment completed the 1-year follow-up assessment.

### 2.3. Clock Drawing Test

The CDT was administered by the same group of neuropsychologists. The participants were given a blank piece of paper and asked to follow a two-step instruction: “First, draw a 10-cm diameter clock face with all the numbers on it. Second, draw hands on the clock to make it read 10:10”. The CDT was scored by the psychologists according to the rating scales developed by Cahn et al. [10]. Briefly, quantitative scores were independently assessed by the 3 raters and consisted of the following three components of the drawing: integrity of the clock face, 0–2 points; presence and sequencing of the numbers, 0–4 points; and presence and sequencing of the hands, 0–4 points. Qualitative scores, which consisted of 8 types of errors (1 point for each present), were subtracted from the quantitative score. A 10-point scale was used, with higher numbers indicating better performance (Table S1). Cronbach’s alpha for the 3 independent raters was 0.980.

### 2.4. Dementia Diagnosis

Dementia was diagnosed according to DSM-5 [22]. Participants who did not satisfy the criteria were assigned to the non-dementia group for analysis. MCI was diagnosed according to Petersen’s criteria [23]. The non-dementia group comprised cognitively normal and MCI participants.

### 2.5. Anti-Dementia Medications

The prescription of anti-dementia medications was confirmed from the chart review during the observation period (between the baseline and follow-up assessments). Medications included donepezil, galantamine, rivastigmine, and memantine. In some cases, more than one medication was prescribed concurrently.

### 2.6. Statistical Analysis

Continuous variables were compared with Student’s *t*-test and categorical variables with the chi-square test. To examine the association of the CDT with progression to dementia, multiple logistic regression analysis was performed, with change in diagnosis from non-dementia to dementia at 1 year as the dependent variable, adjusting for potential confounders among the independent variables. Sensitivity and specificity were calculated using SPSS ver. 25 (IBM, Armonk, NY, USA) before creating the receiver operating curve.

## 3. Results

A total of 163 participants (41.1% men) were enrolled. A flow chart showing participant selection is provided as Figure S1. Mean age of the participants was 76.1 ± 7.1 y. Those who progressed to dementia were 2.5 years older than those who did not. Mean MMSE and CDT scores were 27.4 ± 1.8 and 7.1 ± 2.1, respectively (Table 1). Anti-dementia medications were prescribed in 18.4% of the participants, and there was no significant difference in the use of medications between those who converted to dementia after 1 y and those who did not. Mean MMSE and CDT scores were significantly lower in the group that showed progression to dementia. No statistically significant differences were found in age, sex, education, MMSE, and CDT scores between the participants who were included and those who were excluded for not attending the 1-y follow-up (*n* = 419).

**Figure 1:**
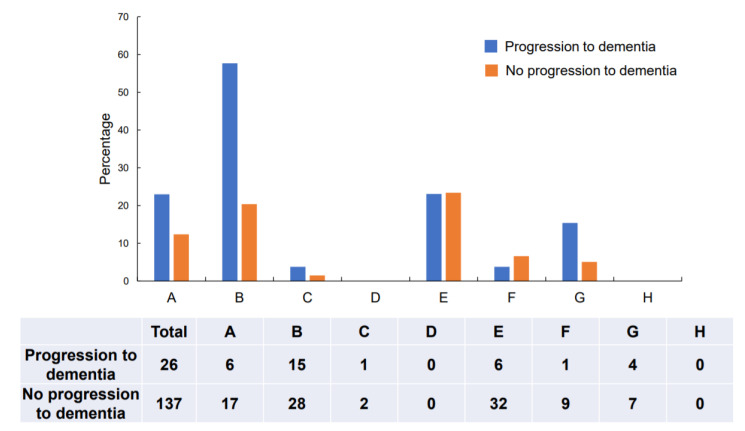
Distribution of error types: Type A: Stimulus-bound response; Type B: Conceptual deficits; Type C: Preservation; Type D: Neglect of left hemispace; Type E: Planning deficits; Type F: Non-specific spatial error; Type G: Numbers written on the outside of the clock; Type H: Numbers written counterclockwise.

Regarding the overall CDT results, 55.8% (91/163) of the participants showed qualitative errors. Conceptual deficits were most frequently observed (27.0%, 43/163), followed by planning deficits (23.3%, 38/163), and stimulus-bound responses (14.1%, 23/163). Conceptual deficits and planning deficits were more frequently observed in the group showing progression to dementia than in the group not showing progression (Figure 1). Typical examples of conceptual deficits, stimulus-bound response, and planning deficits are shown in Figures S2–S4.

A total of 26 (16.0%) participants were diagnosed as having converted to dementia. Diagnoses in this group were dementia of Alzheimer’s type (*n* = 21), vascular dementia (*n* = 2), dementia of Lewy body type (*n* = 2), and frontotemporal dementia (*n* = 1) in the follow-up assessments (374.0 ± 35.5 d after baseline). Multiple logistic regression analysis was performed to determine whether the CDT score based on Cahn et al.’s scoring system or presence of the most frequent error types (conceptual deficits and planning deficits) was associated with progression to dementia. In the analysis, age, sex, education, and anti-dementia medication were included as potential confounders, and the total CDT score (model 1), existence of conceptual deficits (model 2), and planning deficits (model 3) were included as explanatory variables. It was revealed that both the CDT score using Cahn et al.’s scoring system and the existence of conceptual deficits were significantly associated with progression to dementia at 1 y, irrespective of the MMSE score (Table 2). Among the progressed group (*n* = 26), 15 had a conceptual type error in the CDT (sensitivity = 57.7%), and among the non-progressed group (*n* = 136), 28 had a conceptual type error (specificity = 79.6%).

Because the MMSE was independently associated with progression to dementia, we decided to combine the CDT and MMSE. The receiver operating curve of the MMSE scores for predicting progression to dementia is shown in Table S2. It suggested a cut-off score of either 27/26 or 26/25 as being optimal for predicting future progression to dementia by the MMSE score alone. Next, we evaluated the utility of combining the qualitative assessments of conceptual deficits on the CDT with the MMSE score. The sensitivity and specificity of combining the MMSE ≤ 27 (≤26) with conceptual deficits on the CDT were 68.4% and 78.0% (73.3% and 64.9%), respectively (Table 3). Table 4 shows age, sex, years of education, and cognitive assessments in the groups with or without conceptual deficits and results of comparison using Student’s *t*-test. The participants with conceptual deficits on the CDT had poorer cognitive performances as assessed by the ADAS and MMSE.

## 4. Discussion

In this study, we revealed that low scores on the CDT using Cahn et al.’s scoring system showed a significant association with progression to dementia at 1 y in non-demented participants, and the association was independent of the MMSE score. We also found that conceptual deficits on the CDT alone accounted for progression to dementia in non-demented participants. The CDT engages complex cortical networks simultaneously [24,25] as well as mobilizing different cognitive abilities including attention, comprehension, working memory, visual memory, semantic memory, and visuospatial ability [9]. The present study suggests an independent association of the CDT with progression to dementia, which was not necessarily explained by generic cognitive decline as assessed by the MMSE screening instrument. Hence, we attempted to combine these two distinctive measures—the CDT and MMSE—and evaluate their effectiveness in predicting future conversion to dementia in non-demented individuals. In the lower range of the MMSE (<28 or 27/30), conceptual deficit errors on the CDT alone had moderate sensitivity and specificity for predicting progression to dementia. Both the MMSE and CDT are easy to administer and require no special equipment, so they are appropriate for clinical use in primary care settings. Although the CDT alone may not provide sufficient predictive power for detecting progression to dementia, when combined with the MMSE cut-off, the sensitivity and specificity of the CDT for predicting progressions fall within an acceptable range.

Of the 26 cases that converted to dementia at 1 y in this study, 21 (80.7%) developed dementia of Alzheimer’s type. The neural network used to perform the clock drawing task is thought to be closely related to temporal lobe functions, which are impaired in Alzheimer’s pathology [26]. Conceptual deficits, in particular, are associated with semantic deficits, for which temporal lobe dysfunction might be responsible [27]. Cahn et al. proposed their scoring system for screening of Alzheimer’s disease (AD) and found that conceptual deficits increased with progression of AD severity [10].

A previous study exploring the association of error type on the CDT with progression to dementia adopted a different scoring method from that of Cahn et al., and found that substitutional error (e.g., drawing letters/words instead of numbers on the clock), which is considered a conceptual deficit, was significantly associated with progression to dementia [14]. The present study showed that participants with conceptual deficits had reduced memory and general cognitive functions. These findings may explain why conceptual deficits predicted progression to dementia. In a study by Rouleau et al. [27], conceptual deficits increased as AD progressed, and participants with conceptual deficits showed rapid cognitive decline. That finding partly agrees with the present results. Conceptual deficits resulting from loss of both semantic and episodic memory may explain the findings of our study.

Recent technological developments have made it possible to detect AD in the pre-dementia stage. Amyloid and/or tau PET have been developed and proved useful for the early detection of AD [3]. CSF biomarkers have also become candidate predictors for its onset [3]. However, these technologies remain largely impractical in primary care settings because they are very expensive, require special equipment, and CSF sampling is invasive. As an alternative, the CDT is inexpensive and easy to administer. Although its discriminatory power to predict progression to dementia in non-demented participants was not so high in the present study, it could serve as a simple and easy index for such prediction in broader primary care settings. Alternatively, it could be combined with other prediction methods, such as the Cardiovascular Risk Factors, Aging and Dementia (CAIDE) scale [14], which include only non-cognitive risk factors for greater discriminatory power.

We tried to accurately diagnose dementia and non-dementia in this study by using a wide range of cognitive assessments to evaluate the differences between individuals with or without conceptual deficits, and we carefully followed up the participants for at least 1 y. However, we acknowledge some limitations to this study. First, the participants were diagnosed as not having dementia and were required to have two assessments during the course of about 1 y, which meant that many of our patients were excluded, and selection bias should be considered when interpreting our results. Second, the study was conducted at a single institution, and so the results may not be generalizable. Accordingly, in the future, further studies should be conducted at multiple institutions. Third, the CDT administered in this study was evaluated by experienced psychologists, and it should be confirmed whether such evaluations can be performed effectively across primary care settings. Fourth, because sensitivity and specificity were not very high in the present results, the CDT alone may not be adequate for predicting progression from non-dementia status to dementia status. Fifth, because the exact timing of MCI diagnosis was not clear, we could not show the duration of MCI status in participants with MCI.

In conclusion, we found that the qualitative CDT score determined using Cahn et al.’s scoring system in non-dementia subjects was significantly associated with progression to dementia around 1 y later. Conceptual deficits were also significantly associated with progression to dementia. Thus, the CDT may be a useful predictor of progression to dementia that can be easily applied in primary care settings.

## Figures and Tables

**Table 1:** Participant backgrounds.

	All Participants (*n* = 163)	Progression to Dementia (*n* = 26)	No Progression to Dementia (*n* = 137)	*p*-Value
Age, mean ± SD	76.1 ± 7.1	79.0 ± 5.2	75.5 ± 7.3	0.005
Men, *n* (%)	67 (41.1)	9 (34.6)	58 (42.3)	0.520
Education years	12.9 ± 2.8	12.9 ± 3.4	12.9 ± 2.6	0.938
MMSE, mean ± SD	27.4 ± 1.8	26.1 ± 1.6	27.6 ± 1.7	<0.001
CDT, mean ± SD	7.1 ± 2.1	5.6 ± 2.5	7.4 ± 1.9	<0.001
Anti-dementia medications, *n* (%)	25 (18.4)	5 (19.2)	20 (14.6)	0.227

SD: standard deviation; MMSE: Mini-Mental State Examination; CDT: clock drawing test.

**Table 2:** Multiple logistic regression analysis.

	Odds (95% CI) Model 1	*p*-Value	Odds (95% CI) Model 2	*p*-Value	Odds (95% CI) Model 3	*p*-Value
Age	1.071 (0.967–1.119)	0.187	1.088 (0.968–1.141)	0.240	1.062 (0.960–1.174)	0.244
Sex	0.702 (0.191–2.571)	0.593	0.632 (0.159–2.507)	0.514	0.811 (0.238–2.760)	0.737
Education	1.285 (1.015–1.627)	0.037	1.302 (1.017–1.666)	0.036	1.222 (0.983–1.520)	0.071
Anti-dementia medications	2.394 (0.605–9.458)	0.214	2.723 (0.658–11.269)	0.167	2.319 (0.607–80853)	0.218
MMSE	0.640 (0.419–0.976)	0.038	0.594 (0.397–0.889)	0.011	0.533 (0.359–0.792)	0.001
CDT	0.750 (0.569–0.987)	0.040	–	–	–	–
Conceptual deficits	–	–	6.342 (1.825–22.046)	0.004	–	–
Planning deficits	–	–	–	–	1.345 (0.382–4.742)	0.645

CI: confidence interval; MMSE: Mini-Mental State Examination; CDT: clock drawing test.

**Table 3:** Progression to dementia with or without conceptual deficits

**MMSE Score**	**Conceptual Deficits**	**Progression to Dementia**	**No Progression to Dementia**
24 ≤ MMSE ≤ 27	No	6	46
	Yes	13	13
MMSE ≥ 28	No	5	62
	Yes	2	15
	**Conceptual Deficits**	**Progression to Dementia**	**No Progression to Dementia**
24 ≤ MMSE ≤ 26	No	4	27
	Yes	11	10
MMSE ≥ 27	No	7	81
	Yes	4	18

MMSE: Mini-Mental State Examination.

**Table 4:** Cognitive assessments with or without conceptual deficits.

	Total	Without Conceptual Deficits	With Conceptual Deficits	*p*-Value
Participants, *n*	163	120	43	
Age, mean ± SD	76.1 ± 7.1	75.7 ± 7.4	77.0 ± 6.4	0.297
Men, *n* (%)	67 (41.1)	49 (40.8)	18 (41.9)	0.906
Years of education	12.9 ± 2.8	13.1 ± 2.7	12.5 ± 3.0	0.297
CDT	7.1 (± 2.1)	8 (± 1.2)	4.4 (± 1.6)	<0.01
Logical memory I	15.3 (± 7.3)	15.9 (± 7.3)	13.7 (± 7.1)	0.10
Logical memory II	9.1 (± 7.3)	9.6 (± 7.4)	7.7 (± 7.2)	0.14
Word recall immediate	6.1 (± 1.7)	6.3 (± 1.7)	5.5 (± 1.7)	0.01
Word recall delayed	5.5 (± 2.8)	5.8 (± 2.9)	4.7 (± 2.3)	0.01
Verbal fluency category	15.6 (± 4.6)	15.7 (± 4.6)	15.1 (± 4.6)	0.46
Verbal fluency initial letter	9.3 (± 3.8)	9.6 (± 3.9)	8.7 (± 3.4)	0.17
Digit span	13.2 (± 3.3)	13.4 (± 3.4)	12.6 (± 2.9)	0.17
Digit symbol	41.7 (± 12.3)	42.5 (± 12.6)	39.2 (± 11.4)	0.14
Stroop	16.9 (± 13.7)	17.2 (± 14.8)	16.6 (± 10.0)	0.81
TMT-A	63 (± 26.5)	61.5 (± 27.3)	68.1 (± 23.8)	0.18
TMT-B	162.6 (± 82.0)	161.9 (± 86.3)	166.3 (± 68.8)	0.78
ADAS-J Cog	8.3 (± 4.9)	7.6 (± 3.9)	10.3 (± 6.8)	0.02
MMSE	27.4 (± 1.8)	27.6 (± 1.7)	26.8 (± 1.9)	<0.01

Data are shown as the mean (±standard deviation). CDT: clock drawing test; TMT: trail making test; ADAS-J Cog: Alzheimer’s Disease Assessment Scale—Cognitive component, Japanese version; MMSE: Mini-Mental State Examination.

## Supplementary Materials

The following are available online at https://www.mdpi.com/2077-0383/9/9/2850/s1, Figure S1: Flow chart of participant selection, Figure S2: Typical presentations of conceptual deficits in clock drawing tests, Figure S3: Typical presentations of stimulus-bound response in CDT, Figure S4: Typical presentations of planning deficits in CDT, Table S1: Cahn’s rating scale, Table S2: Sensitivity and specificity by Mini-Mental State Examination cut-off
