# Supplementary material for: Association of the Qualitative Clock Drawing Test with Progression to Dementia in Non-Demented Older Adults
Source: J Clin Med. 2020 Sep 3;9(9):2850. doi: 10.3390/jcm9092850 (PMC7563645; doi:10.3390/jcm9092850)
Supplement: Supplementary file 1 [file jcm-09-02850-s001.pdf]

Journal of
Clinical Medicine

MDPI

Supplementary Materials for

Association of the Qualitative Clock Drawing Test With Progression to Dementia in Non-Demented Older Adults

Hiroyuki Umegaki[a*], Yusuke Suzuki[b], Yosuke Yamada[a], Hitoshi Komiya[a], Kazuhisa Watanabe[a], Masaki Nagae[a], Masafumi Kuzuya[a]

[a] Department of Community Healthcare and Geriatrics, Nagoya University Graduate School of Medicine, 65 Tsuruma-cho, Showa-ku, Nagoya, Aichi 466-8550, Japan

[b]Centre for Community Liaison and Patient Consultations, Nagoya University Hospital, 65 Tsurumai-cho, Showa-ku, Nagoya, Aichi 466-8560 Japan

*Corresponding author: Dr. Hiroyuki Umegaki

Department of Community Healthcare and Geriatrics, Nagoya University Graduate School of Medicine, 65 Tsuruma-cho, Showa-ku, Nagoya, Aichi 466-8550, Japan

Tel.: +81-52-744-2364; Fax: +81-52-744-2371

Email: umegaki@med.nagoya-u.ac.jp

Figure S1 flow chart of participant selection

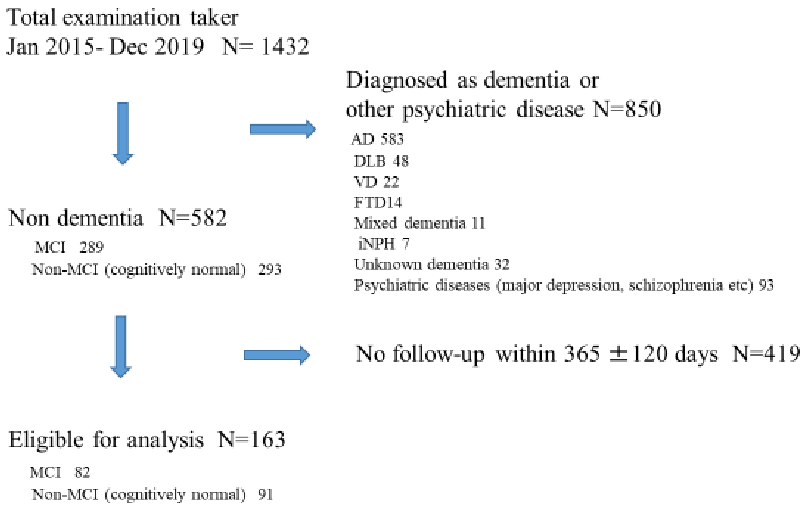

Figure S2 Typical presentations of conceptual deficits in CDT
conceptual deficits are defined as miss-presentation of the clock itself or the time

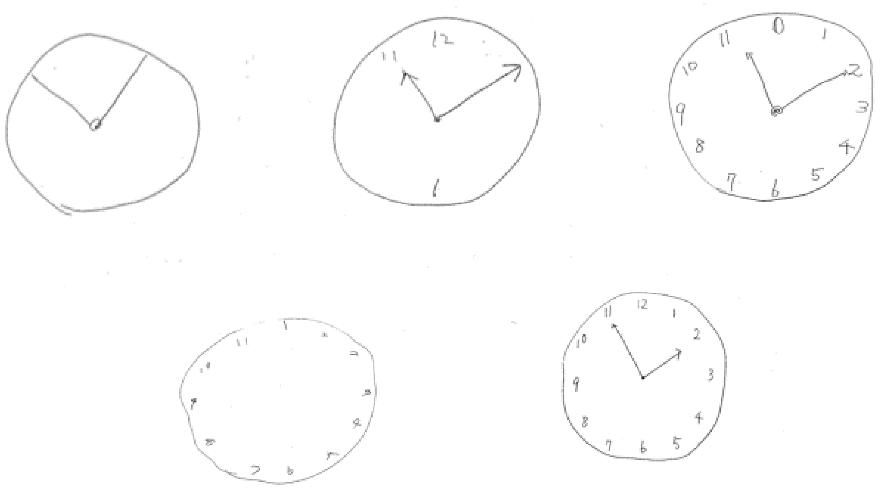

Figure S3 Typical presentations of stimulus-bound response in CDT

stimulus-bound response is tendency of the drawing to be dominated or guided by a single stimulus

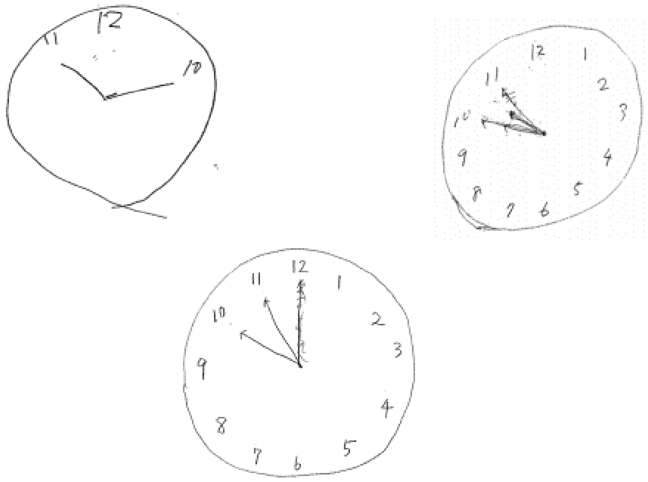

Figure S4 Typical presentations of planning deficits in CDT

planning deficits is represented by gaps before 12, 3, 6 or 9

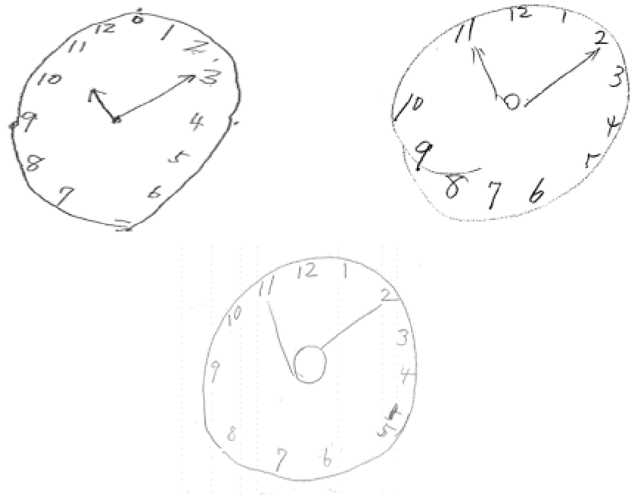

Table S1. Cahn's rating scale

I Quantitative CDT score (maximum 10 points). Assesses the presence and correctness of the following clock attributes: clock face (0– 2 points), placement of the hands (0–4 points), and placement of the numbers (0–4 points).

II Qualitative CDT score (maximum 8 points). Assesses the presence or absence of the following errors (1 point for each present).

A. Stimulus-bound response: the tendency of the drawing to be dominated or guided by a single stimulus
B. Conceptual deficit: reflects a loss or deficit in accessing knowledge of the attributes, features, and meaning of a clock
C. Perseveration: the continuation or recurrence of an activity without an appropriate stimulus
D. Neglect of left hemispace: all attributes of the clock are written on the right side of the clock face
E. Planning deficit: this error type is represented by gaps before 12, 3, 6 or 9
F. Nonspecific spatial error: a deficit in the spatial layout of numbers, without any specific pattern in spatial disorganization
G. Numbers written on the outside of the clock: numbers written either around the perimeter of the circle or on the circle itself
H. Numbers written counterclockwise: arrangement of the numbers with ‘12’ at the top of the clock face and then continuing around in a counterclockwise fashion

Table S2. Sensitivity and specificity by MMSE cut-off point

Cut-off point	Sensitivity	Specificity
29.5	0.139	100
28.5	0.365	0.962
27.5	0.562	0.731
26.5	0.730	0.577
25.5	0.869	0.423
24.5	0.964	0.231

Area under the curve = 0.742

© 2020 by the authors. Submitted for possible open access publication under the terms and conditions of the Creative Commons Attribution (CC BY) license (http://creativecommons.org/licenses/by/4.0/).
